# HIV status and mortality of surgical inpatients in rural Zimbabwe: A retrospective chart review

**DOI:** 10.4102/sajhivmed.v20i1.812

**Published:** 2019-01-24

**Authors:** Pascal Migaud, Michael Silverman, Paul Thistle

**Affiliations:** 1 Department of Infectious Diseases and Gastroenterology, Vivantes Auguste-Viktoria-Klinikum, Germany; 2 Department of Infectious Disease, Faculty of Medicine, Western University, Canada; 3 Department of Obstetrics and Gynaecology, Karanda Mission Hospital, Zimbabwe; 4 Department of Obstetrics and Gynaecology, Faculty of Medicine, University of Toronto, Canada

## Abstract

**Background:**

People living with HIV treated with antiretroviral therapy (ART) are now living longer and thus many are requiring surgical procedures. For healthcare resource planning, it would be helpful to better understand the prevalence of HIV in surgical patients, the types of surgery HIV-positive patients are undergoing and whether HIV status impacts mortality.

**Objective:**

The goal of this study was to determine the prevalence of HIV in surgical inpatients and the extent of ART coverage, as well as to assess any differences between HIV-positive and HIV-negative patients in type of surgery undergone and in-hospital mortality at Karanda Mission Hospital, Mount Darwin, Zimbabwe.

**Method:**

A 1-year retrospective chart review was undertaken to collect clinical and demographic data for adult (excluding maternity cases) and paediatric surgical inpatients including age, sex, type of surgery, HIV status, CD4+ counts and, if patient was HIV-positive, whether he or she was taking ART.

**Results and conclusion**: Charts for 1510 surgical inpatient stays were reviewed. HIV prevalence among the adults was higher than that in the general population in Zimbabwe in 2016 (23.2% vs. 14.7%). There was no significant difference in inpatient mortality between the HIV-negative group and the HIV-positive group. Within the group of patients with malignancies, people living with HIV were significantly younger than uninfected patients (mean age 50.5 vs. 64.4 years; *p* < 0.01). There were correlations between HIV and certain malignancies. Thus, in addition to AIDS-defining illnesses, clinicians must be alert to squamous cell carcinoma and oesophageal, anal and penile cancers in HIV-positive patients.

## Introduction

With antiretroviral therapy (ART) extending the life expectancy of patients with human immunodeficiency virus (HIV), more HIV-positive patients are undergoing surgical procedures. One review in sub-Saharan Africa (SSA) from 2016 reported that HIV prevalence in surgical patients was higher than in the general population.^1^ Because of the lack of universal testing, many patients are not aware of their status; the rate of testing in surgical inpatients has been reported to be as low as 10.0% – 12.0%.^2^

In comparison to HIV-negative patients, HIV-infected patients are more likely to have to undergo surgery to treat infectious complications, such as abscesses or Fournier’s gangrene.^3^ Besides the well-known AIDS-defining malignancies such as Kaposi’s sarcoma, non-Hodgkin’s lymphoma (NHL) or invasive cervical cancer, which were added to the Centers for Disease Control’s list of AIDS-defining conditions in 1993, there is growing evidence that there is a link between other malignancies and HIV.^3^ Lung, liver and anal cancers are more common in HIV-positive patients and often present at a more advanced stage or at an earlier age compared to the general population.^4^ To plan for surgical services and training of healthcare professionals, we need to better understand the types of surgery HIV-positive patients are likely to require and how HIV status impacts mortality.

In 2016, the prevalence of HIV in adults (aged 15–49 years) in Zimbabwe was 14.7%, which was among the five highest rates in SSA.^5^ Antiretroviral therapy coverage has been dramatically increased, but the 90-90-90 goal of the Joint United Nations Programme on HIV/AIDS (UNAIDS) and the World Health Organization (WHO) – which stipulates that by 2020, 90% of individuals would be aware of their HIV status, 90% of those who are HIV-positive should be on ART and 90% of those treated should have achieved viral suppression – has not yet been reached.^6^ HIV prevalence among surgical patients and its impact on outcomes has not been documented in Zimbabwe.

In this study, we aimed to determine the prevalence of HIV in surgical inpatients and the extent of ART coverage, as well as assess any differences between HIV-positive and HIV-negative patients in type of surgery undergone and in-hospital mortality at Karanda Mission Hospital, Mount Darwin, Zimbabwe.

## Methods

Karanda Mission Hospital is a 150-bed rural community hospital in northern Zimbabwe where over 4000 surgeries are performed annually.

A retrospective chart review of surgical inpatient records from 01 January 2016 to 31 December 2016 was conducted to collect clinical and demographic data including age, sex, type of surgery performed, HIV status, CD4+ counts and, if patient was HIV-positive, whether he or she was taking ART. In addition, histological information was collected from a separate record that contains pathology results provided by Lancet Laboratories Harare, Zimbabwe. Missing CD4+ counts were extracted from the locally available IT system at Karanda Mission Hospital. All inpatient surgeries performed on children (defined as those below 15 years of age) and adults, excluding maternity cases, were included. Each visit corresponded to a separate entry in the study.

Before analysing the data, all cases were anonymised. General descriptive statistical analysis and further testing for statistical significance with cross tables, chi-square, phi and Cramer’s V was performed. Additionally, we used Monte Carlo simulation and univariate analysis. All statistical analysis was performed with IBM SPSS Statistics 22.

## Ethical consideration

There is no local institutional ethics committee; thus, ethics approval for this retrospective chart review was granted by the administrators at the hospital.

## Results

### Patient characteristics

A total of 1510 surgical inpatient cases were identified (Table 1). Fifty-one patients (3.4%) presented more than once to the hospital: one patient underwent surgery three times and the remaining 50 patients were admitted twice. Eight hundred and ninety patients (61.0%) were male. The average age was 46.2 years (range 1–97, median 49). Forty-seven patients (3.1%) died in hospital.

**TABLE 1:** Patient characteristics.

Variable	HIV+				HIV-				Status unknown (test not performed)			
		
	*n*	%	mean	s.d.	*n*	%	mean	s.d.	*n*	%	mean	s.d.
**Age (years)**	45.56	-	1–87	14.62	48.9	-	1–96	24.67	39.76	-	1–97	31.35
**Total**	177	43.5†	-	-	1044	61.9†	-	-	288	70.1?	-	-
Children (< 15)	4	75.0†	-	-	140	67.1†	-	-	114	71.9†	-	-
Adult (15–49)	110	35.5†	-	-	356	46.6†	-	-	41	70.7†	-	-
Older adults (> 49)	63	55.6†	-	-	548	70.4†	-	-	133	68.4†	-	-
**Diabetes**	3	1.7	-	-	53	5.1	-	-	7	2.4	-	-
**HTN**	17	9.6	-	-	215	20.6	-	-	46	16.0	-	-
**RPR performed**	118	66.8	-	-	238	22.8	-	-	19	6.6	-	-
Positive	4	2.5	-	-	22	2.4	-	-	5	1.7	-	-

RPR, rapid plasma reagin test for syphilis; HTN, arterial hypertension; s.d., standard deviation.

† , male.

**FIGURE 1:**
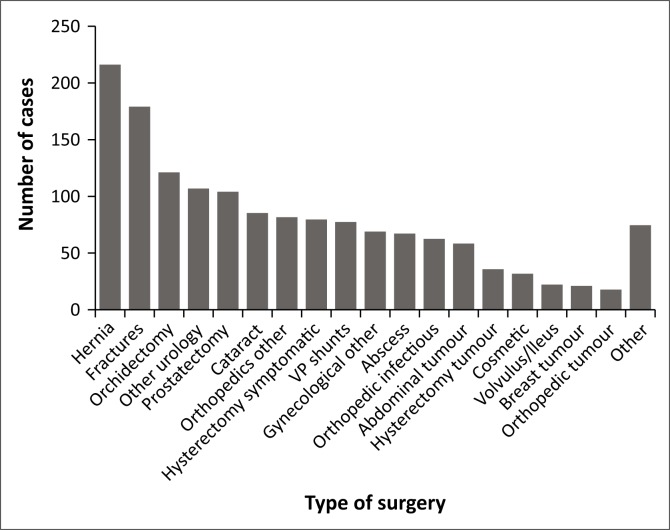
Type of surgery.

We established 32 different categories of surgery (Figure 1). Hernia repair was the most common procedure (216 cases; 14.3%), followed by fracture repairs (179; 11.9%), orchidectomies as palliative therapy in metastasised prostate cancer (129; 8.0%) and other urology cases and prostatectomies (107, 7.1%, and 104, 6.9%, respectively).

### Histology

**FIGURE 2:**
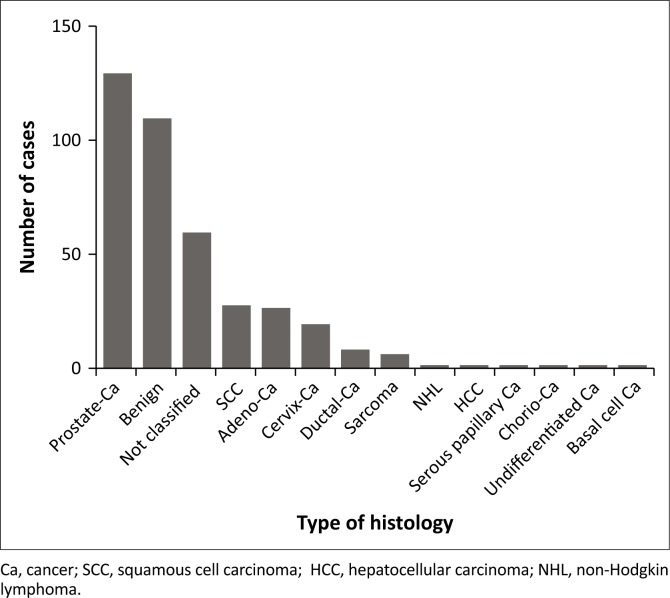
Type of histology.

Histology was usually not performed for the bilateral orchidectomy (BLO) cases. The diagnosis of metastasised prostate cancer was therefore either based on clinical presentation and PSA levels and/or on histology provided by the patient. Two hundred and sixty-eight cases for histology were sent to Lancet Laboratories Harare. The most common findings were benign (*N* = 109) or not classifiable (*N* = 59). Including the BLO cases, prostate cancer was by far the most common malignancy, accounting for 129 cases (58.4%), followed by squamous cell carcinomas (all sites 27; 12.2%), adenocarcinoma (all sites 26; 11.8%) and cervical cancer (19; 8.6%) (Figure 2).

### HIV and other comorbidities

Of the patients, 1045 (69.0%) had tested negative for HIV at the time of hospitalisation or during the preceding three months, while 288 (19.1%) of the patients did not have a documented valid test at the time of hospitalisation, that is, no test had been performed or the last test was performed over three months prior. Further, 11.9% of the patients had a positive HIV test or were known to be HIV-positive.

For further statistical analysis we excluded all patients without a valid test. In the group of patients between 15 and 49 years of age, 110 (23.3%) were HIV-positive. This group, usually defined as the adult reproductive-aged group by UNAIDS and WHO, accounted for 62.0% of all HIV patients in this cohort. Four children (2.8% of all children) were HIV-positive. In the group of adults over 49 years, 63 (10.3%) were HIV-infected.

In the paediatric group, 43.4% did not have a valid HIV test, compared to 9.3% and 17.9% in the adult group and the group over 49 years of age, respectively.

Twenty-one patients were newly diagnosed with HIV because of provider-initiated testing and counselling (PITC), although this approach was not fully initiated at Karanda Mission Hospital until the beginning of 2017. Seven (33.0%) of these patients were started on ART before discharge and 18 (90.0%) were started on cotrimoxazole prophylaxis.

Of the HIV-positive patients, 156/177 (88.1%) were receiving ART; in two cases it was impossible to establish whether ART was part of the patient’s treatment. Five (2.8%) of the patients were on second-line treatment. Moreover, 88.1% of the patients were taking cotrimoxazole prophylaxis. A recent CD4+ count was available for 117 of the 177 patients (66.0%). The mean value was 413 cells/µL (range 0 cells/µL–1510 cells/µL, median 371 cells/µL, SD 287 cells/µL). Thirty-one (26.5%) had a CD4+ count below 200 cells/µL.

Of the 47 patients who died during hospitalisation, nine (19.0%) were HIV-positive.

There was no significant difference in mortality between the HIV-negative group and the HIV-positive group (*p* = 0.39).

**FIGURE 3:**
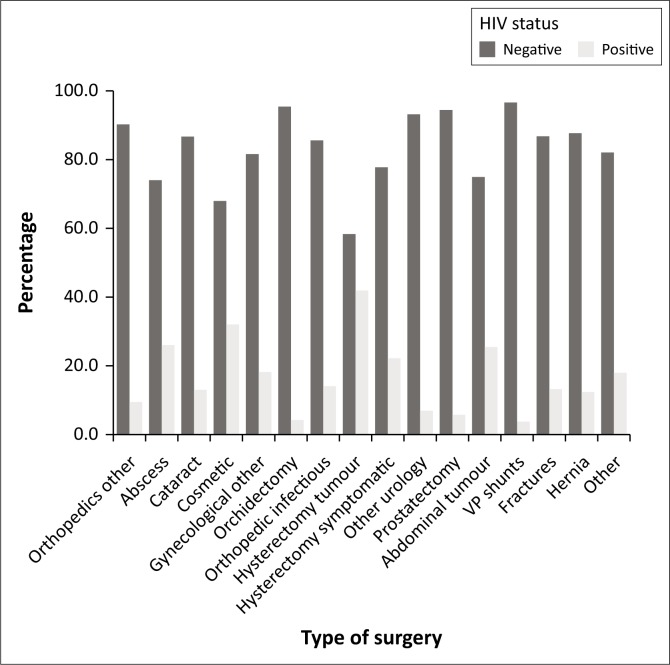
HIV status by type of surgery.

There was a significant correlation between the type of surgery and HIV status. HIV-positive patients were more likely to undergo surgery for hysterectomy because of suspected cancer, laparotomy because of suspected gastrointestinal malignancy or procedures for abscesses and plastic surgery. In contrast, this group was less likely to undergo surgery for cerebral shunts, prostatectomy or BLO (Figure 3; Table 2).

**TABLE 2:** Association between HIV and certain types of surgeries.

Type of surgery	Rate of HIV		Likelihood to be	*p*	Mean age
	
	*N*	%	HIV-positive		
Hysterectomy tumour	15	41.7	2.9-fold	< 0.001	51.14
Cosmetic	8	32.0	2.2-fold	0.012	33.20
Abscess	13	26.0	1.8-fold	0.018	36.44
Gastrointestinal tumour	14	25.5	1.75-fold	0.018	53.78
Cerebral shunts	2	3.7	0.26-fold	0.02	4.96
Prostatectomy	5	5.9	0.4-fold	0.019	70.09
Bilateral orchidectomy	4	4.3	0.3-fold	0.004	71.26

### HIV and malignancies

Thirty-seven (20.2%) of the patients with histologically proven malignancies were HIV-positive. When only patients in the group aged 15–49 years were analysed, 19 (46.3%) of those with malignancies were HIV-positive. Twelve (63.2%) of the patients with cervical cancer were HIV-positive, 14 (51.9%) of the patients with squamous cell carcinoma were HIV-positive and three (13.6%) of the patients with adenocarcinoma were HIV-positive. In contrast 9 (4.1%) of all patients with prostate cancer were HIV-positive.

**FIGURE 4:**
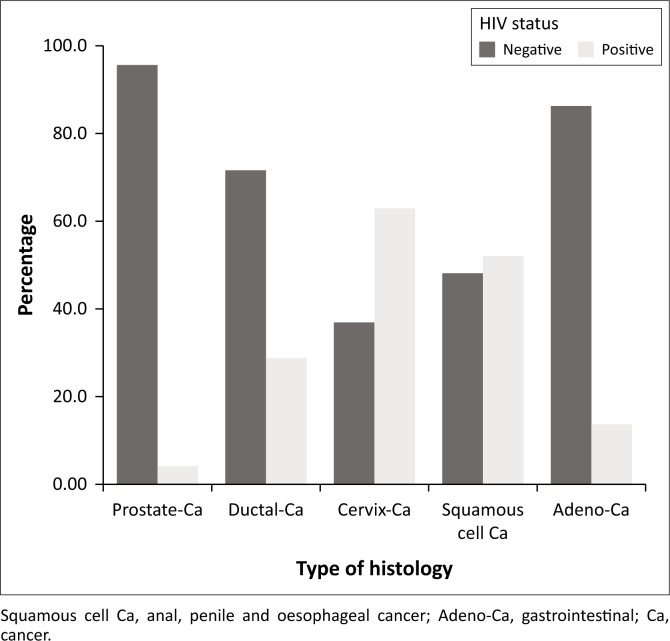
HIV status by histology.

Chi-square test showed a highly significant correlation between HIV status and certain types of histology with a *p*-value of *p* < 0.001 and a strong association with a Cramer’s V value of 0.6 (Figure 4). Being HIV-positive was associated with a higher likelihood of having cervical cancer or squamous cell carcinoma (3.16- and 2.54-fold greater, respectively; *p* < 0.001).

In contrast, patients with prostate cancer were 4.9 times less likely to be HIV-positive (*p* < 0.003). There was a significant difference between the mean age of the prostate cancer group and the remainder of the patients (71.2 vs. 45.7 years of age, respectively; *p* < 0.05).

Regarding the other diagnoses, trends but no statistically significant correlations were found.

Within the group of patients with malignancies, people living with HIV were significantly younger than uninfected patients (mean age 50.5 vs. 64.4 years; *p* < 0.01). Excluding all patients with prostate cancer (*N* = 86), HIV-positive patients were still younger (48.8 vs. 52.1), but the difference was not statistically significant (*p* = 0.126).

## Discussion

### Epidemiology

HIV prevalence among the adult surgical patients reviewed was higher than that in the general population in Zimbabwe in 2016 (23.2% vs. 14.7%).^6^ Similar findings have been reported in studies from South Africa and Malawi.^3,7^ Women were over-represented in the HIV-positive adult group (64.5%). This may be because Karanda Mission Hospital is well known as a centre for gynaecologic and obstetric surgery. Distribution of the different age groups was similar to that reported in the UNAIDS Gap report from 2016.

### Testing and therapy

The Zimbabwe Demographic and Health Survey of 2010–2011 showed that only 57.0% of women and 36.0% of men aged 15–49 years had ever been tested for HIV.^8^ The follow-up report from 2015 showed an increase in testing from the previous survey, with 49.0% of women reporting being tested in the last 12 months compared to 34.0% in 2011. Among men, this increased from 21.0% in 2011 to 36.0% in 2015.^9^

Previous studies have reported very low testing rates among surgical patients, ranging from 10.0% to 50.0%.^3,10^ In our cohort, 80.9% had a valid and documented HIV test. In 2007, the WHO recommended a PITC approach for all patients encountering the health system.^11^ Several studies have demonstrated the positive effect of this approach.^12,13,14^ The Zimbabwe Ministry of Health introduced PITC in 2007, which stated that anyone presenting to any level of healthcare institution should be offered HIV testing regardless of the purpose of their visit.^15^ Twenty-one patients were newly diagnosed with HIV during our study period, eight of whom presented with indicator conditions, such as cervical cancer, anal cancer or vaginal warts and abscesses. Eighteen of these patients were started on cotrimoxazole prophylaxis and only seven began ART. These findings show that PITC for all patients, even those without an indicator condition, is both feasible and warranted. With the new test-and-treat approach, where patients start their ART in absence of an opportunistic infection the same day as the test, the numbers of patients who start ART in hospital will certainly increase.

Of the HIV patients in our cohort, 88.1% were on ART; only 2.8% of these were on second-line treatment. While this is heartening, to reach the 90-90-90 goal, PITC will have to be scaled up further.^16^ In Zimbabwe, viral load measurements are not routine. Therefore, it remains unclear if the low number of patients on second-line treatment was because of the success of first-line ART or the lack of virological monitoring.

Interestingly, all the patients on ART were also on cotrimoxazole prophylaxis independent of the CD4+ count (mean CD4+ count 413 cells/µL and 73.5% > 200 cells/µL). According to the Zimbabwean guidelines, this is recommended. It appears to be acceptable in an area that has a high prevalence of malaria and bacterial infections.

### HIV, type of surgery and outcome

HIV-positive patients in our cohort were significantly more likely to undergo hysterectomy because of suspected cancer, plastic surgery and abscess drainage or debridement. These findings are not surprising as cervical cancer is an AIDS-defining condition^17^ and HIV-positive patients are known to have HPV-associated genital warts,^18^ which accounted for most of the plastic surgery in our cohort. The higher prevalence of abscesses among HIV-positive cohorts has been demonstrated in several previous studies.^8,11,19^

There was no difference in in-hospital mortality between HIV-positive and HIV-negative surgical inpatients. This contrasts with a study in Malawi, which, in a small cohort, found a higher mortality rate in HIV-positive patients.^17^

In several studies from SSA, most of them in the pre-ART era, a significant difference in surgical outcomes and complications was not found.^20^ However, there seems to be evidence for a higher mortality rate in HIV-positive patients undergoing emergency surgery.^21^

### HIV and malignancies

In addition to the known AIDS-defining malignancies, HIV-infected patients are also more likely to suffer from certain cancers.^5^ In our study, there was a strong correlation between HIV and certain malignancies. In the adult age group 15–49 years, 46.3% of the patients with proven malignancies were HIV-positive. Unsurprisingly, HIV prevalence was highest among cervical cancer patients (63.2%). In addition to this, however, we found a higher prevalence (51.4%) in the squamous cell carcinoma group and a 2.54-fold greater likelihood of having HIV. Oesophageal, skin and penile cancers were also common in HIV-positive patients. The association with HPV and penile cancer is well established.^22^ While ART has decreased the incidence of NHL, more cases of oesophageal cancer and stomach cancer are being diagnosed. Whether this is also because of HPV or an independent risk factor, such as is the case for lung cancer, remains unclear.^23^ This may be because HIV-positive patients encounter healthcare workers more often through accessing ART and may therefore have a lower threshold for seeking help at a health facility, leading to better diagnosis of disease.

Within our cohort, few men undergoing surgery for prostate cancer were HIV-positive and the average age of the group with prostate cancer was 71.2 years. Thus, the average age of HIV-positive patients with malignancies was significantly younger than that of HIV-negative patients with malignancies.

Our study had several limitations. Outpatients, and therefore most cases of surgery and maternity cases, were excluded. Older men were overrepresented. Likewise, not all histology results were available. Also, because of the lack of access to routine laboratory investigations, no biochemical profile was available for the patients. Furthermore, information about hepatitis, TB or HPV coinfections was not available in most of the cases. Because this was a retrospective chart review, only inpatient mortality could be captured. In this setting of rural Zimbabwe, it would be very difficult to follow-up patients to determine 30-day mortality.

In conclusion, this study shows a higher HIV prevalence among adult surgical inpatients than in the general population. We demonstrated that testing and treatment is well established in this specific rural Zimbabwean hospital. Interestingly, in-hospital mortality was not higher in HIV-positive patients compared to HIV-negative patients. There were correlations between HIV and certain malignancies. Thus, in addition to AIDS-defining illnesses, clinicians must have a high index of suspicion for squamous cell carcinoma and oesophageal, anal, penile cancers in HIV-positive patients. In countries experiencing a high prevalence of HIV, surgical training programmes and surgical units in hospitals must anticipate the types of surgeries that HIV-positive patients are likely to require, such as excision of malignancies, and provide adequate training, staffing and facilities to meet the needs for the future.
